# A Comparative Study of the Bacterial Community in Denitrifying and Traditional Enhanced Biological Phosphorus Removal Processes

**DOI:** 10.1264/jsme2.ME13132

**Published:** 2014-06-24

**Authors:** Xiao-Mei Lv, Ming-Fei Shao, Chao-Lin Li, Ji Li, Xin-lei Gao, Fei-Yun Sun

**Affiliations:** 1 Harbin Institute of Technology Shenzhen Graduate School, Shenzhen, Guangdong, 518055, China; 2 Shenzhen Key Laboratory of Water Resource Utilization and Environmental Pollution Control, Shenzhen, Guangdong, 518055, China; 3 Shenzhen Public Technological Service Platform for Urban Waste Energy Regeneration, Shenzhen, Guangdong, 518055, China

**Keywords:** EBPR, denitrifying phosphorous removal, microbial community, pyrosequencing

## Abstract

Denitrifying phosphorus removal is an attractive wastewater treatment process due to its reduced carbon source demand and sludge minimization potential. Two lab-scale sequencing batch reactors (SBRs) were operated in alternating anaerobic-anoxic (A-A) or anaerobic-oxic (A-O) conditions to achieve denitrifying enhanced biological phosphate removal (EBPR) and traditional EBPR. No significant differences were observed in phosphorus removal efficiencies between A-A SBR and A-O SBR, with phosphorus removal rates being 87.9% and 89.0% respectively. The community structures in denitrifying and traditional EBPR processes were evaluated by high-throughput sequencing of the PCR-amplified partial 16S rRNA genes from each sludge. The results obtained showed that the bacterial community was more diverse in A-O sludge than in A-A sludge. Taxonomy and β-diversity analyses indicated that a significant shift occurred in the dominant microbial community in A-A sludge compared with the seed sludge during the whole acclimation phase, while a slight fluctuation was observed in the abundance of the major taxonomies in A-O sludge. One *Dechloromonas*-related OTU outside the 4 known *Candidatus* “Accumulibacter” clades was detected as the main OTU in A-A sludge at the stationary operation, while *Candidatus* “Accumulibacter” dominated in A-O sludge.

Enhanced biological phosphorus removal (EBPR) is considered to be the most cost-effective and environmentally friendly technology to meet the increasingly stringent discharge standard of wastewater treatment plants (2). In the EBPR process, polyphosphate-accumulating organisms (PAO) are favored and enriched for phosphorus removal through alternating anaerobic-oxic (A-O) conditions. Under the anaerobic condition, PAO are capable of storing organic substances (particularly volatile fatty acids (VFAs)) as intracellular poly-β-hydroxyalkanoates (PHA) through the release of orthophosphate and degradation of glycogen. Under the subsequent oxic condition, the stored PHA is oxidized to produce energy for the uptake of excessive amounts of orthophosphate, biomass growth, and regeneration of glycogen. By discharging polyphosphate-enriched excess sludge, phosphorus is ultimately removed from the wastewater.

In addition to the alternating anaerobic-aerobic condition, phosphorus can also be removed under anaerobic-anoxic (A-A) cycling with nitrate or nitrite as an electron acceptor (1, 20, 25, 34). The organisms responsible for anaerobic-anoxic phosphorus removal are referred as denitrifying PAO (DPAO). The internal carbon source (typically PHA) is utilized for both phosphorus removal and denitrification in the same process; therefore, the demand for an often-limiting carbon source can be reduced, with approximately 50% of the carbon source being saved. Moreover, the denitrifying phosphorus removal process can save aeration by approximately 30% and minimize excess sludge (21, 30).

The activities of DPAO have been widely studied and reported since the discovery of denitrifying phosphorus removal (4, 10, 32). However, it remains controversial whether the same organisms are responsible for phosphorus removal under oxic and anoxic conditions. DPAO were hypothesized to be the same organisms as PAO, and the different activities observed under different environments were only attributed to the inducing conditions (35, 39). Unlike PAO, which can use only oxygen as an electron acceptor, DPAO can utilize oxygen or nitrate for phosphorus removal (6, 23, 38). Previous studies also demonstrated that DPAO were a fraction of PAO (1, 20, 41).

Recently developed culture-independent approaches have identified the *Rhodocyclus*-related organism, *Candidatus* “Accumulibacter” as one of the most important PAO in EBPR systems (13, 18, 26). Based on an elaborate online survey and subsequent phylogenetic analysis of reported 16S rRNA gene sequences from full scale wastewater treatment plants (WWTPs) and bench scale reactors (22, 27), four clades (namely Acc-SG1, Acc-SG2, Acc-SG3 and Acc-SG4) were assigned by Kim *et al.* (13). *Candidatus* “Accumulibacter” may have also adapted to different ecological environments and exhibits various physiological properties similar to DPAO (5, 11, 12, 15). In addition to the clades of *Candidatus* “Accumulibacter”, *Dechloromonas-*related bacteria had been detected in several EBPR processes operated under partially anoxic conditions (15, 17, 19). However, whether they function as PAO remains controversial. According to Kong’s study (19), a *Dechloromonas-*like organism (Bet135 FISH probe positive) was found to have a polyphosphate-accumulating phenotype by culture-independent methods. Although another *Dechloromonas*-like organism (Dech453 FISH probe positive) was abundant in A-A-O SBR, it was predicted to only reduce nitrate to nitrite in the reactor, and *Candidatus* “Accumulibacter” acted as the real PAO in this system (15). Due to their wide distribution in wastewater treatment-related environments and disputed functions, the phosphorous removal activity of *Dechloromonas*-related bacteria needs to be elucidated in more detail.

In the present study, two sequencing batch reactors (SBRs) were operated under alternating anaerobic-anoxic (A-A) or anaerobic-oxic (A-O) conditions with nitrate or oxygen as the electron acceptor in order to establish strict denitrifying phosphorous removal and conventional EBPR environments, respectively. A comparative study of the phosphorus removal performance of the two SBRs was firstly reported. The whole community structure, dominant taxonomy, and community dynamics during acclimation were systematically compared with the aid of high-throughput sequencing to detect DPAO. The further characterization of the denitrifying phosphorous removal sludge community and identification of DPAO in this study has provided an insight into denitrifying phosphorus removal and process efficiency.

## Materials and Methods

### Reactor operation

**Fig. 1:**
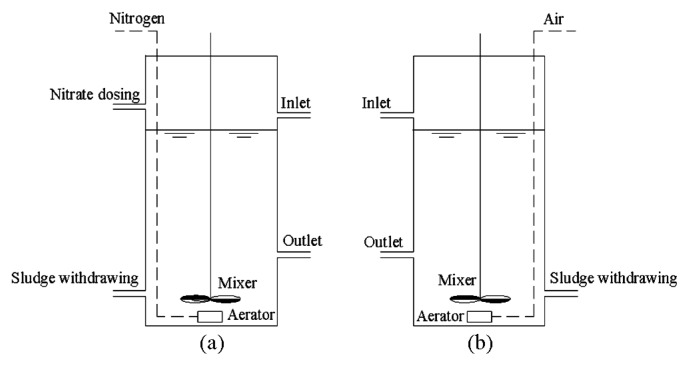
Configuration of the two lab-scale phosphorous removal SBRs.

Two parallel sequencing batch reactors with an effective working volume of 10.9 L were configured in the present study (Fig. 1) for continuous operation in order to conduct phosphorus removal studies. The A-A SBR was operated under an alternating anaerobic-anoxic condition for the acclimation of denitrifying phosphate removal sludge, while the A-O SBR was operated under an anaerobic- oxic condition for the acclimation of traditional phosphate removal sludge as a control reactor for the A-A SBR.

Both SBRs were inoculated with the sludge from a full-scale sewage WWTP with an A^2^/O (anaerobic-anoxic-aerobic) process and operated at room temperature with an operation cycle of 8 h. Each of the above cycles consisted of five stages: filling (0.25 h), anaerobic (2.5 h), anoxic or oxic (4.0 h), settling (1.0 h), and withdrawing (0.25 h). Mixers were operated during the anaerobic and anoxic (or oxic) stages for better mixing at a rate of 40 r min^−1^. In the A-A reactor, nitrate was dosed continuously for the initial 1.0 h of the anoxic stage with a peristaltic pump (Lange, BT-100). The initial nitrate concentration was designed at 20 mg L^−1^ based on the influent COD concentration (the C: N ratio was approximately 7.5:1), and the dosing flow of the nitrate solution was timely adjusted (±5%–10%) to achieve an effluent nitrate concentration of 2–7 mg L^−1^ during the whole operation. These parameters were used to satisfy complete anoxic phosphorus uptake and avoid excessive nitrate at the beginning of the anaerobic phase. In the A-O reactor, air was aerated at flow rate of 0.3 m^−3^ h^−1^ during the oxic stage with an aerator to supply oxygen for aerobic phosphorus removal sludge. All of the above operations were automatically controlled with time controllers. Moreover, pH was uncontrolled except that the initial pH was adjusted to approximately 7.0 at the beginning of the anaerobic stage. The SRT of the sludge in the A-A and A-O SBRs were 15–20 d and 10–15 d, respectively.

At the end of each cycle, 7.2 L of supernatant was exchanged with an equal volume of synthetic wastewater. Each liter of the wastewater contained 200 mg CH_3_COONa, 14 mg NH_4_Cl, 20 mg NaH_2_PO_4_, and 0.3 mL trace element solution, which consisted (1 L) of 10 g EDTA, 0.12 g ZnSO_4_·7H_2_O, 0.12 g MnSO_4_·7H_2_O, 1.0 g FeCl_3_, 0.03 g CuSO_4_·5H_2_O, 0.15 g CoCl_2_·6H_2_O, 0.06 g (NH_4_)_2_Mo_4_O_13_·2H_2_O, 0.15 g H_3_BO_3_, and 0.18 g KI. Real primary settled sewage wastewater was also mixed with the synthetic wastewater at a ratio of 1:10 by volume to maintain microbial diversity.

### Chemical analysis

Sampling was conducted regularly at the end of the anaerobic and anoxic (or oxic) stages as well as the synthetic influent for the concentration analysis of COD, NH_4_^+^-N, NO_3_^−^-N, NO_2_^−^N, and PO_4_^3−^-P. COD was measured using the potassium dichromate method and PO_4_^3−^-P was quantified by the molybdate colorimetric method using a spectrophotometer (Shimadzu, UVmini-1240). The N-component including NH_4_^+^-N, NO_3_^−^N, and NO_2_^−^-N were measured automatically by colorimetric methods with a Cleverchem200 (DeChem-Tech.Gmbh, Germany).

### DNA extraction, PCR amplification, and High-throughput Sequencing

Total DNA were extracted from sludge samples using the FastDNA^®^ SPIN Kit for Soil (MP Biomedicals, Illkirch, France). The PCR primer set (33) of 967F (CAACGCGAAGAACCTTACC) and 1046R (CGACAGCCATGCANCACCT) was chosen to target the V6 hypervariable region of the bacterial 16S rRNA gene. Each 30 μL PCR reaction system contained 0.75 μL of MightyAmp^®^ DNA Polymerase (Takara, Dalian, China), 15 μL of 2×Buffer, 1.5 μL of each primer, and 20–50 ng of genomic DNA. The amplification was conducted in an i-Cycler (BioRad Laboratories, CA, USA) under the following thermo steps: initial denaturation at 98°C for 2 min, followed by 28 cycles at 98°C for 20 s, 55°C for 20 s, 68°C for 1 min, and a final extension step at 68°C for 5 min. In order to minimize the impact of potential early-round PCR errors, three parallel amplifications of each DNA were conducted simultaneously and then mixed together for further analysis. The quality of the amplification products was examined by agarose (2.0%) gel electrophoresis. Lastly, PCR amplicons were purified with the quick Midi Purification Kit (Qiagen) and quantified using spectrometry (NanoDrop-1000).

Barcodes that allowed sample multiplexing during sequencing were incorporated into both the forward and reverse primers for V6 amplicon sequencing (31). The amplicons from different samples were then mixed together by ensuring equal mass concentrations in the final mixture, which was sent out for library construction and sequencing on the Illumina-HiSeq 2000 at the Beijing Genomics Institute (BGI) of Shenzhen with the strategy of paired-end sequencing (2×100 bp).

Independent metagenomic-based community profiling of one sludge DNA sample from the A-A SBR on day 274 (March 22, 2013) was carried out in parallel with V6 amplicon-based quantification to validate the accuracy of community analysis based on 16S rRNA gene partial amplicons. Briefly, DNA was mechanically fragmented to an enrichment size of –170 bp. The DNA fragments were then gel purified and quality checked. Recycled DNA was used for shotgun library construction, which was finally sequenced on an Illumina HiSeq 2000 platform using the Paired End 100 bp sequencing strategy at the BGI of Shenzhen.

### Bioinformatic analysis

Routine amplicon sequence processing, including primer removal, low quality sequence screening, and sample sorting, were firstly carried out using the Pyrosequencing Pipeline of the Ribosomal Database Project (RDP). The qualified sequences of each sample were then denoised to remove sequencing-induced errors by the shhh.seqs command in Mothur. Removal of the archaeal sequence was based on the RDP classifier (Version 2.5) and carried out by a self-written C++ program. OTUs were obtained by processing the incorporated cluster file through another two self-written C++ program, and OTU-based principle coordinate analysis (PCoA) and cluster analysis (CA) were performed using PAST software, as described by Zhang *et al.* (40). The microbial diversity indices in terms of Chao 1, Chao 2, and ACE (Abundance-based Coverage Estimator) as well as the Good’s coverage were calculated using the EstmateS tool. The comprehensive taxonomy information at different levels was confirmed via Global Alignment for Sequence Taxonomy (GAST).

In the metagenomic-based analysis, low quality reads screening was performed by PRINSEQ (http://sourceforge.net/), then pairedend (PE) reads were pair-aligned by FLASH (http://www.cbcb.umd.edu/software/flash) to screen 10–50 bp overlapping PE reads and subsequently assemble them into 150–190 bp iTags. Finally, iTags were processed with MetaPhlAn (http://huttenhower.sph.harvard.edu/metaphlan/) for comprehensive population profiling of the sludge microbial community.

### Accession number

The V6 amplicon sequences of 16S rRNA reported in this study have been deposited into the short reads archive (SRA) database of NCBI (http://www.ncbi.nlm.nih.gov/) with the accession number of SRR768434. The metagenomic data sets of this study have been deposited in the MG-RAST server (http://metagenomics.anl.gov/) and the accession number was 4524971.3.

## Results and Discussion

### Performance of the two SBRs

**Fig. 2:**
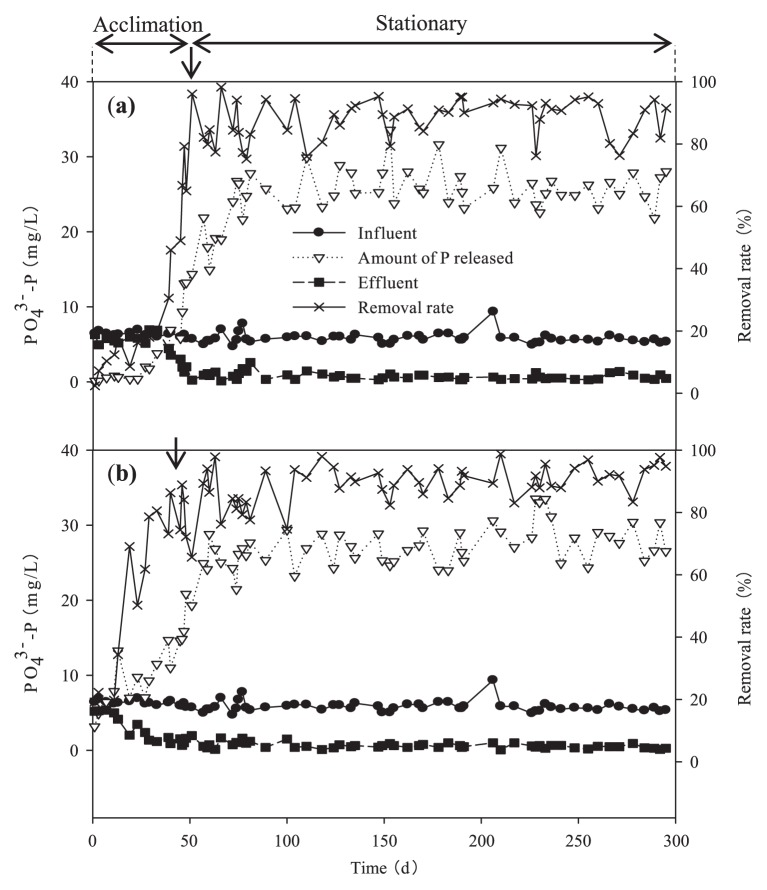
Overall phosphorus removal during a 295-day operation.

The whole profiles of the PO_4_^3−^-P concentration and phosphorus removal rate from inoculation until stationary operation were shown in Fig. 2. The concentration of PO_4_^3−^-P in the influent was controlled at approximately 6.0 mg L^−1^ during the entire study, and it took 5 L and 40 d for the A-A and A-O SBRs to achieve stationary operation, respectively. In the A-A SBR, the phosphorus removal rate increased gradually and stabilized at approximately 87.9%. The acclimation period in the A-O SBR was shorter, which was attributed to more PAO in the seed sludge being inherited due to the similar redox conditions between the A-O SBR and full-scale A^2^/O WWTP environment, and the phosphorus removal rate in the A-O SBR stabilized at approximately 89.0%. Thus, after acclimation, the two SBRs were continuously operated until day 295 to evaluate the operation stability of denitrifying phosphorus removal. During the entire stationary operation, the PO_4_^3−^-P concentration in the effluent was 0.225–1.76 mg L^−1^ (averaged at 0.728 mg L^−1^) for the A-A SBR and 0.072–1.61 mg L^−1^ (averaged at 0.664 mg L^−1^) for A-O SBR, respectively. These results indicated the stability of denitrifying phosphorus removal process. No significant differences were observed in the phosphorus removal efficiencies between the two SBRs, which was consistent with the findings by Kapagiannidis *et al.* (12).

### Sample selection for microbial community structure analysis

The sludge samples used in the microbial community analysis were summarized in Table 1. Firstly, the seed sludge from WWTP (noted as D0) was collected to obtain initial microbial information on the two SBRs before acclimation. Sludge samples were collected from the two SBRs at both the acclimation and stationary phases, according to the phosphorus removal efficiency and amount of anaerobic phosphorus released, to demonstrate the succession of the microbial community during the acclimation phase. As shown in Table 1, sludge samples taken on days 9 and 35 were selected to represent the acclimation phase and samples on days 55 and 76 were selected to represent the stationary phase.

### Microbial diversity of different EBPR sludges

As shown in Table 2, a total of 19,331–28,419 raw sequences were generated for the nine samples through high-throughput sequencing. After filtering out low quality and archaeal sequences and denoising, 8,373–11,572 sequences remained and were considered to be the effective sequences for different samples. In order to achieve a fair comparison at the same depth, the sequences of all nine samples were normalized to 8,373, which was the smallest of these samples, for downstream analysis.

The diversity indices, namely, OTUs, Chao1, Chao2, ACE, Bootstrap, and GOOD’s coverage based on the above-described normalized sequences at both the 3% and 6% cut-off levels, were summarized in Table 2. On the basis of OTUs number, the microbial diversity of the seed sludge was the highest because it was taken from the full scale WWTP, which dealt with more complex pollutants in municipal wastewater than the synthetic influent. Moreover, sludge in the A-O SBR was more diverse than that in the A-A SBR, and this may have been because of the anaerobic-anoxic condition of the A-A SBR laying additional burden on the microbial community, thereby resulting in a less diverse community after the acclimation phase. Similar results to the OTUs analysis were observed with Chao1, Chao2, ACE, and Bootstrap as well as the rarefaction curve (Fig. S1).

### β-diversity

Two independent methods, Cluster analysis (CA) and Principal coordinate analysis (PCoA) were adopted to compare the similarity of the sludge microbial community among the two SBRs and seed sludge. Based on the abundances of the OTUs at the 3% cut-off and phylum levels, Cluster analysis (Fig. S2) and Principal coordinate analysis (Fig. S3) showed that sludge samples from the A-A SBR were clustered together as one group and samples from the A-O SBR formed another group, which indicated the diverse microbial community in denitrifying phosphorus removal sludge and conventional phosphorus removal sludge. Moreover, after acclimation under different operation modes, the community structure in the A-O SBR was more similar to the seed sludge (A^2^/O process) than the denitrifying A-A SBR sludge. This may have been because of the similar redox conditions between the A-O SBR and real A^2^/O process.

The identity of denitrifying phosphorus removal bacteria remains controversial. Based on the results of the above β-diversity analysis, the introduction of the A-A condition may have induced a shift in the bacterial community structure in EBPR, which has been reported in a previous FISH study (15).

### Dominant taxonomy

**Fig. 3:**
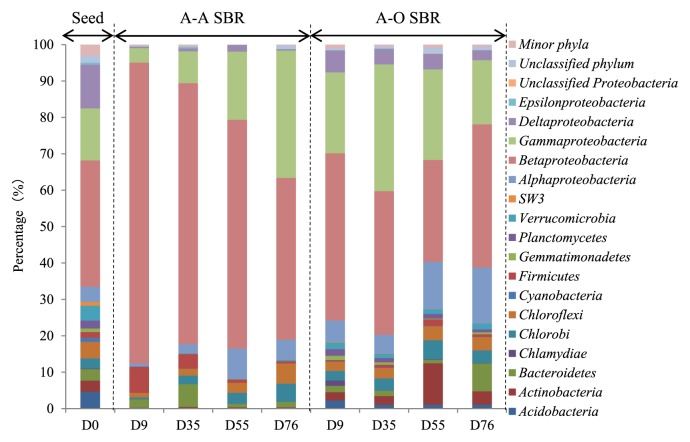
Abundances of different phyla and classes in *Proteobacteria* in the 9 sludge samples.

The 8,373 selected effective bacterial sequences in each sample were assigned to different taxa (from phylum to genus) via Global Alignment for Sequence Taxonomy (GAST), as shown in Fig. 3.

Similar to the community structure of globally distributed WWTP (28, 36, 40), *Proteobacteria* were the most abundant phylum in all samples, accounting for 65.8%–91.8% of the total bacterial sequences. Other dominant phyla were *Chloroflexi* (1.27–5.54%, averaged at 3.20%), *Chlorobi* (0.47–5.27%, averaged at 3.16%), *Bacteroidetes* (0.97– 7.64%, averaged at 2.89%), and *Actinobacteria* (0.16–11.4%, averaged at 2.64%), followed by several other major (average abundance>1%) phyla including *Firmicutes* (1.97%), *Acidobacteria* (1.16%) and *Verrucomicrobia* (1.15%). In addition, *Acidobacteria*, *Actinobacteria*, and *Verrucomicrobia* were the major phyla in the seed and A-O sludges, with abundances in the A-A sludge being markedly low (averaging at 0.09%, 0.24%, and 0.12% respectively).

Within *Proteobacteria*, *Betaproteobacteria* was the most dominant class in all nine samples, followed by *Gammaproteobacteria*, *Alphaproteobacteria*, and *Deltaproteobacteria* in the eight samples from the two SBRs, while the abundant classes in the seed sludge were *Gammaproteobacteria*, *Deltaproteobacteria*, and *Alphaproteobacteria*. Moreover, the abundances of *Epsilonproteobacteria* were only 0–0.59% (average of 0.16%) in the nine sludge samples. In addition to the four classes of *Proteobacteria*, there were some other dominant shared classes (abundance>1% occurred in at least 50% of samples) including *Chlorobia*, *Actinobacteria*, *Anaerolineae*, *Sphingobacteria*, and *Flavobacteria*.

The abundance of *Betaproteobacteria* decreased from 82.7% to 44.6% in sludge samples collected from the A-A SBR during the 76-day operation, while the abundance of *Gammaproteobacteria* increased by nearly 9-fold from 3.99% to 34.9%. The abundances of *Alphaproteobacteria* and *Deltaproteobacteria* also slightly increased after the acclimation phase. As for the A-O SBR, the abundances of *Betaproteobacteria* and *Gammaproteobacteria* fluctuated within a small range during acclimation, while that of *Alphaproteobacteria* slightly increased and *Deltaproteobacteria* decreased.

Apart from the important phosphorous removal relevant functional groups, which have been discussed below, the abundances of filamentous organisms that cause sludge bulking and foaming were also compared. The abundances of filamentous genera including *Caldilinea*, *Gordonia*, *Microthrix*, *Nostocoida*, *Trichococcus*, and *Zoogloea* (10) in denitrifying and aerobic phosphorus removal sludges were 1.76% and 0.681%, respectively, in samples collected on day 76. Their abundances were slightly higher in denitrifying phosphorus removal sludge, which may have been the reason for its slightly worse sludge-setting ability than that of aerobic phosphorus removal sludge.

These results indicated that microbial communities changed during the acclimation phase, especially in the A-A SBR, which led to a significant shift in the microbial community as a result of the A-A operation.

### Validation of V6 amplicon-based microbial population quantification by independent metagenomic analysis

In the present study, taxonomic profiling of sludge microbial communities mainly relied on the sequencing and classification of the PCR-amplified 16S ribosomal RNA gene V6 region. Owing to its universality in prokaryotes and the availability of comprehensive reference databases, the 16S rRNA gene is regarded as a powerful phylogenetic marker; however, it has been criticized for biases introduced by copy-number variations, inconsistent amplification efficiencies, and heterogeneity when targeting different 16S rRNA regions (http://www.nature.com/nmeth/journal/vaop/ncurrent/full/nmeth.2693.html#ref4).

Due to the possible bias in PCR-based quantification, an independent metagenomic-based analysis MetaPhlAn was applied to evaluate the microbial community analysis of sludge samples (March 22, 2013) collected from the A-A SBR based on V6 amplicon. MetaPhlAn is a newly developed analysis platform for profiling microbial communities that uses unique clade-specific marker genes as a BLAST database selected from more than 3,000 reference microbial genomes (29). Due to its current cost disadvantage over 16S rRNA gene amplicon-based methods, MetaPhlAn is rarely utilized to routinely profile community shift dynamics; however, its strength in comprehensive and accurate taxonomic profiling have been demonstrated for representative samples analysis ranging from the human microbiome (24) to wastewater treatment plant (3).

**Fig. 4:**
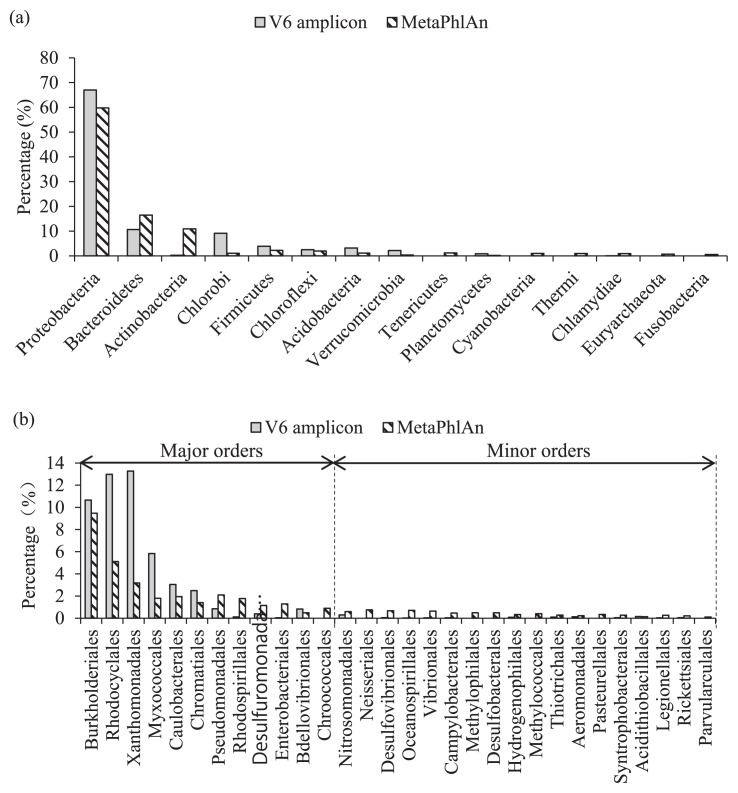
Abundances of phyla (a) and orders within *Proteobacteria* (b) of the A-A SBR sludge (day 274) by V6 amplicon and MetaPhlAn methods.

Fig. 4(a) shows the distribution pattern of both methods at the phylum level. Seven major phyla (including *Proteobacteria* and *Bacteroidetes*) were ranked as the top phyla by both methods. These top phyla accounted for 97.7% and 92.9% of the total community, as revealed by V6 amplicon and MetaPhlAn respectively. The minor difference observed in the phylum level distribution may have been due to the lack of clade-specific marker genes in the environmental metagenome (soil, sediment, or sludge).

As was demonstrated in the present and previous studies (28, 36, 40), *Proteobacteria* dominated in all sludge samples; therefore, the abundances of different orders within *Proteobacteria* were comprehensively compared. Fig. 4(b) showed the abundances of covered orders within *Proteobacteria* (8) by the two methods. In spite of certain abundance fluctuations, both methods revealed similar distribution characteristics of the major and minor orders.

These results supported the effectiveness of the current V6 amplicon-based quantitative analysis of microbial communities.

### DPAO in a strict A-A system

**Fig. 5:**
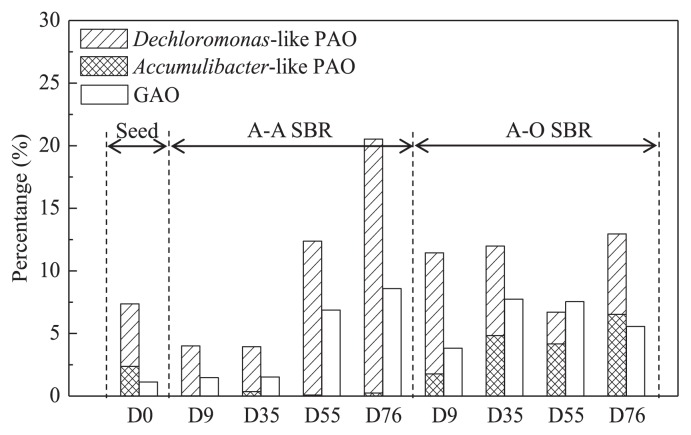
Dynamics of the *Accumulibacter*-like PAO (OTU-196), *Dechloromonas*-like PAO (OTU-1), and GAO (OTUs-87 and 312) populations in A-A and A-O systems.

An important task was to identify the PAO (or DPAO) responsible for phosphorus removal in the current A-A and A-O SBRs. The traditional strategy for this relied on sequence similarity comparisons against already identified PAO. As previously described, *Candidatus* “Accumulibacter” was the most frequently reported and solely well accepted PAO in the activated sludge system. Thus, local BLAST (Basic Local Alignment Search Tool) was firstly attempted using a self-made local database containing all 41 typical *Candidatus* “Accumulibacter” sequences that had summarized in a phylogenetic tree by Kim *et al.* (13). The results shown in Fig. 5 indicated that 2.36% of the sequences in the seed sludge could be assigned to *Candidatus* “Accumulibacter”- related sequences (OTU196). Regarding the A-O sludge, the abundances of *Candidatus* “Accumulibacter”-related sequences were 1.77–4.83% and 4.16–6.53% for the acclimation and stationary phases. These two ratios were slightly below the average reported PAO percentages of 6–22% (7, 43), but were still acceptable. However, only 0.02–0.36% (0.16% on average) of sequences in the A-A sludge were significantly similar to the known *Candidatus* “Accumulibacter”, which was in contrast to the similar phosphorus removal efficiencies between A-A and A-O SBR. If we accepted that the nearly negligible 0.16% *Candidatus* “Accumulibacter”- related population could not have performed the observed luxury phosphorus uptake flux, the only explanation was that a novel DPAO must exist in the current A-A system. Therefore, to identify this novel DPAO, another strategy emphasizing the isolation source of the related NCBI Genbank sequences was attempted, as illustrated in Fig. S4 and Table S1. After screening, OTU-1 came to the fore based on its significant abundance, as shown in Fig. 5, and frequent detection records in very similar phosphorous-removing SBR systems (9, 42) and full-scale EBPR (19, 37). Based on all these findings, OTU-1 was proposed as the main DPAO responsible for phosphorus removal in the current strict A-A system.

**Fig. 6:**
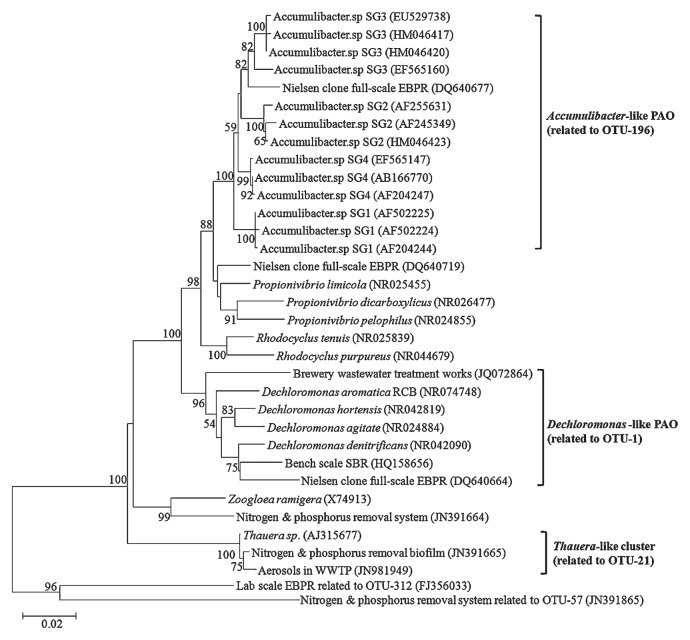
Phylogenetic tree.

The phylogenetic relationship between OTU-1, the *Rhodocyclus*-related 4 clades (13) of *Candidatus* “Accumulibacter”, and the other important species within the family *Rhodocyclaceae* was shown in Fig. 6. All selected environmental sequences related to OTU-1 fell in a separate cluster that was related more to *Dechloromonas denitrificans*. OTU-1 shared similar sequences in the V6 region to *Dechloromonas*-related organisms from a full-scale EBPR clone (DQ640664) identified by Kong *et al.* (19). In their study, this group of organisms, which matched probe Bet135, was shown to be positive for both polyphosphates and PHA staining. Thus, the Bet135-defined organism was proposed to be putative DPAO and was suspected to behave similarly to *Candidatus* “Accumulibacter” in terms of the uptake of substrates and storage of PHA and polyphosphate (19). This group of PAO may play an important role in WWTP because many identical sequences that originated in the phosphorus removal sludge, *e.g.* HQ158656 and JQ072864, have been deposited to the NCBI GenBank. Unfortunately, their potential phosphorus removal function has not been properly annotated. In the present study, the *Dechloromonas*-related organism was further detected as putative DPAO in an independent environment, and was able to tolerate long-term strict A-A operation (Fig. 5), which was attributed to its competitive advantage over *Candidatus* “Accumulibacter”. Thus, our results supported and also extended previous findings (15, 19).

One clear advantage of our identification strategy was its independence on known PAO sequence information. The ability of organisms to cycle phosphorus for energy during EBPR was exhibited by organisms from a range of different phylogenies inclusive of, but not restricted to, members of *Gammaproteobacteria*, *Betaproteobacteria*, and *Actinobacteria* (26). In our approach, each abundant OTU was checked by the isolation source of their close relatives in the NCBI. This was the reason why one *Dechloromonas-*related organism outside of the four known clades could be successfully targeted. In the current case, the uptake and storage behavior of phosphorous by the organism had already been comprehensively characterized by Kong’s pioneering work. Otherwise, the candidates identified by our approach should be strictly examined using *in situ* techniques.

### Dynamics of PAO and the glycogen-accumulating organisms (GAO) population in A-A and A-O systems

The dynamics of PAO, including the *Accumulibacter*-like PAO and *Dechloromonas*-like PAO population, during the whole acclimation phase was shown in Fig. 5. Only minor abundance variations were observed in PAO in the A-O SBR (6.06%–12.0%), and this was attributed to a similar redox condition to that of A^2^O/WWTP. Regarding the A-A SBR, PAO abundances decreased in the initial acclimation phase (3.98% on average) due to the pressure associated with the anaerobic-anoxic condition. After acclimation for approximately two months, the PAO percentage gradually increased to 16.9% on average at the stable phase, which was consistent with the demonstrated phosphorus removal performance.

The presence of GAO is known to potentially compete with PAO due to its uptake of VFA under anaerobic conditions, but not the accumulation of polyphosphate under aerobic conditions (14, 16). GAO was quantified by Local BLAST of each sample with a local database containing 48 known GAO sequences (Table S2). As shown in Fig. 5, the abundance of GAO in the seed sludge was low (0.93%) as a result of operation control in WWTP. In the acclimation phase, the percentage of GAO in A-O SBR was markedly higher than that in A-A SBR, with an average percentage of 4.65% and 1.22%, respectively. In the stable phase, the abundances of GAO in the A-O and A-A SBRs averaged 6.19% and 5.26%, respectively, which suggested that the abundance of GAO was higher under the comparatively singular conditions of anaerobic-anoxic or anaerobic-oxic than full-scale WWTP.

## Conclusion

The quantification of PAO and GAO in the present study provided a simple and convenient approach for monitoring the important functional microbial population in wastewater treatment systems.

## Supplementary Information



## Figures and Tables

**Table 1:** Sludge samples selected for microbial community analysis

Origin	Seed	A-A SBR				A-O SBR			
			
Phase	Inoculation	Acclimation phase		Stationary phase		Acclimation phase		Stationary phase	
Code	SEED-D0	AA-D9	AA-D35	AA-D55	AA-D76	AO-D9	AO-D35	AO-D55	AO-D76
Sampling time	Jun.20	Jun.28	Jul.24	Aug.15	Sep.05	Jun.28	Jul.24	Aug.15	Sep.05
PO_4_^3−^-P release (mg L^−1^)	/	0.67	4.63	23.9	25.4	7.14	10.6	24.9	26.9
PO_4_^3−^-P removal (%)	/	11.3	25.6	82.1	84.7	18.3	73.2	89.3	84.5

**Table 2:** Microbial diversity of the 9 sludge samples

Code	Sequence			3% cut-off						6% cut-off					
		
	Raw	Effective	NO after normalization	OTUs	Chao1 mean	Chao2 mean	ACE mean	Bootstrap mean	GOOD’s coverage	OTUs	Chao1 mean	Chao2 mean	ACE mean	Bootstrap mean	GOOD’s coverage
SEED-D0	20290	8979	8373	1784	3672.8	1701	3493.4	1701	88.21	1468	2392.9	1384	2338.7	1384	91.76
AA-D9	20967	9113	8373	579	1103.8	525	1005.2	525	95.98	416	680.8	354	638.5	354	97.40
AO-D9	26104	10031	8373	1100	2298.1	1096	2378.8	1096	92.76	846	1282.6	777	1264.2	777	95.45
AA-D35	24325	9416	8373	868	1898.0	773	1797.0	773	93.49	623	1063.8	537	1023.3	537	95.95
AO-D35	9501	8373	8373	1012	1624.5	907	1776.9	907	93.26	764	1047.8	666	1089	666	95.90
AA-D55	28232	10861	8373	626	1220.9	555	1147.6	555	95.52	401	652.4	346	585.4	346	97.65
AO-D55	25193	10792	8373	1023	1780.9	925	1851.9	925	92.99	727	927.0	648	958.1	648	96.38
AA-D76	24447	10022	8373	747	1348.6	680	1476.1	680	94.83	519	765.2	450	798.9	450	97.00
AO-D76	28278	11572	8373	1003	1952.6	930	2089.6	930	92.99	744	1119.6	668	1120.4	668	95.71
