# A Comparative Study of the Bacterial Community in Denitrifying and Traditional Enhanced Biological Phosphorus Removal Processes

**DOI:** 10.1264/jsme2.ME13132e

**Published:** 2014-12

Xiao-Mei Lv^1,2^, Ming-Fei Shao^1,2,3^, Chao-Lin Li^1,2^, Ji Li^1,2,3*^, Xin-lei Gao^1,2^, and Fei-Yun Sun^1,2,3^

^1^*Harbin Institute of Technology Shenzhen Graduate School, Shenzhen, Guangdong, 518055, China;* ^2^*Shenzhen Key Laboratory of Water Resource Utilization and Environmental Pollution Control, Shenzhen, Guangdong, 518055, China; and* ^3^*Shenzhen Public Technological Service Platform for Urban Waste Energy Regeneration, Shenzhen, Guangdong, 518055, China*

Volume 29, No. 3, Page 261–268, 2014

Incorrect:

Xiao-Mei Lv^1,2^, Ming-Fei Shao^1,2,3^, Chao-Lin Li1^2^, Ji Li^1,2,3*^, Xin-lei Gao^1,2^, and Fei-Yun Sun^1,2,3^

^*^ Corresponding author. E-mail: liji98@tsinghua.org.cn;

Tel: +86–0755–2603–2692; Fax: +86–0755–2670–3651.

Correct:

Xiao-Mei Lv^1,2^, Ming-Fei Shao^1,2,3*^, Chao-Lin Li^1,2^, Ji Li^1,2,3**^, Xin-lei Gao^1,2^, and Fei-Yun Sun^1,2,3^

^*^ Corresponding author. E-mail: mfshao@hitsz.edu.cn

Tel: +86–0755–2603–2692; Fax: +86–0755–2670–3651.

^**^ Corresponding author. E-mail: liji98@tsinghua.org.cn;

Tel: +86–0755–2603–2692; Fax: +86–0755–2670–3651.

This correction has been completed on the electronic file of the present paper available at J-STAGE.

