# Supplementary material for: A Comparative Study of the Bacterial Community in Denitrifying and Traditional Enhanced Biological Phosphorus Removal Processes
Source: Microbes Environ. 2014 Jun 24;29(3):261–8. doi: 10.1264/jsme2.ME13132 (PMC4159037; doi:10.1264/jsme2.ME13132)
Supplement: Supplementary file 1 [file 29_261_s1.pdf]

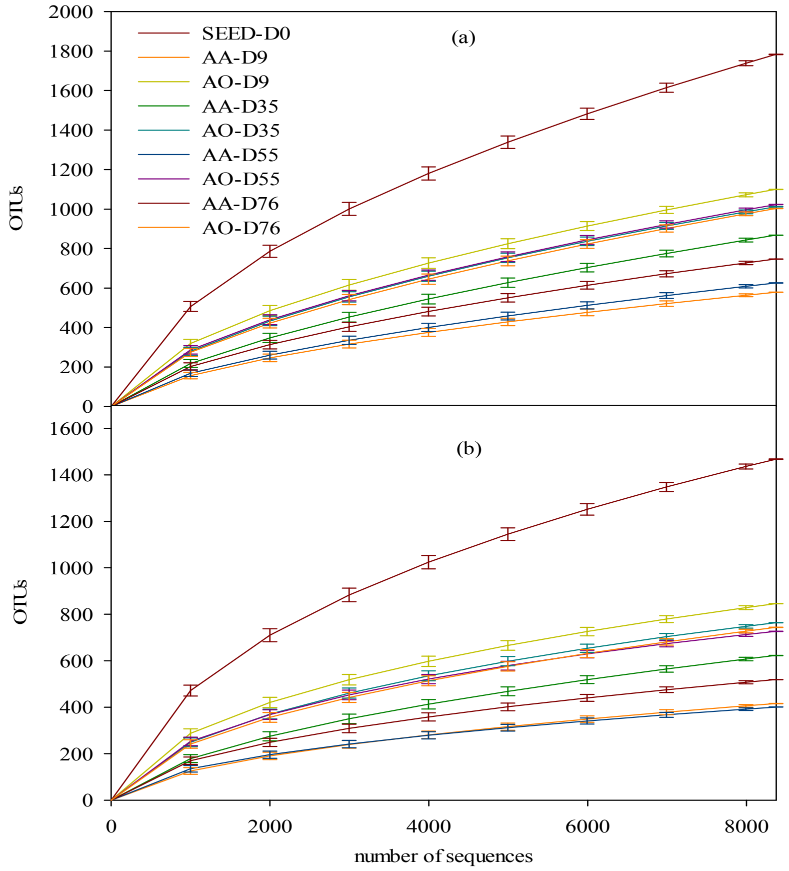


**Fig. S1.** Rarefaction curves of the 9 samples at cutoff levels of 3% (a) and 6% (b). The rarefaction curve, plotting the number of observed OTUs as a function of the number of sequences, was computed using RDP Pyrosequencing Pipeline Rarefaction tool.

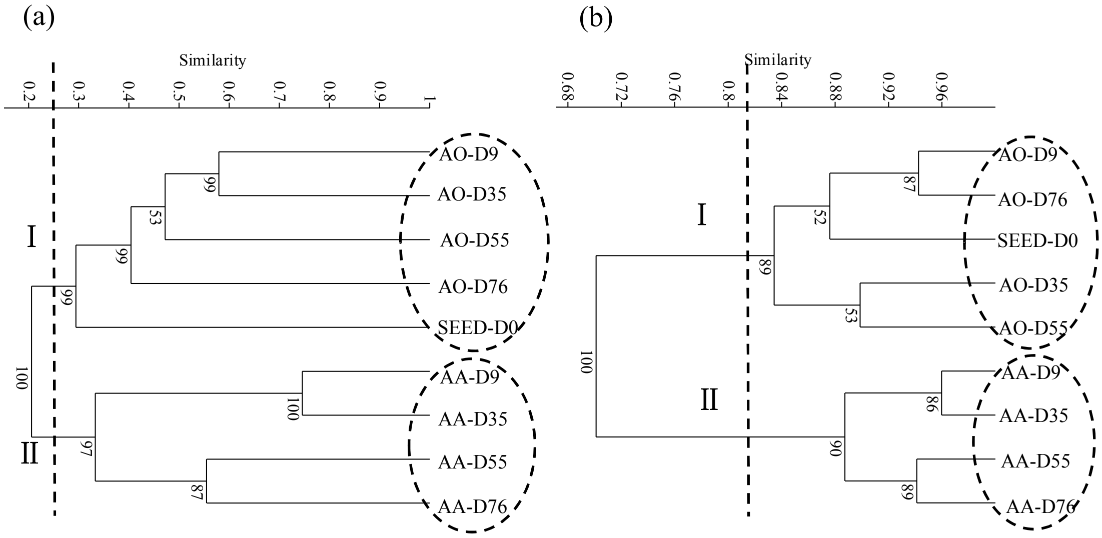


**Fig. S2.** Cluster analysis based on Bray-Curtis distances of 9 samples. (a) at 3% cutoff OTU level; (b) at Phylum level. The dot lines show the similarity cutoff levels to cluster the 9 sludge samples into distinct A-A and A-O groups.

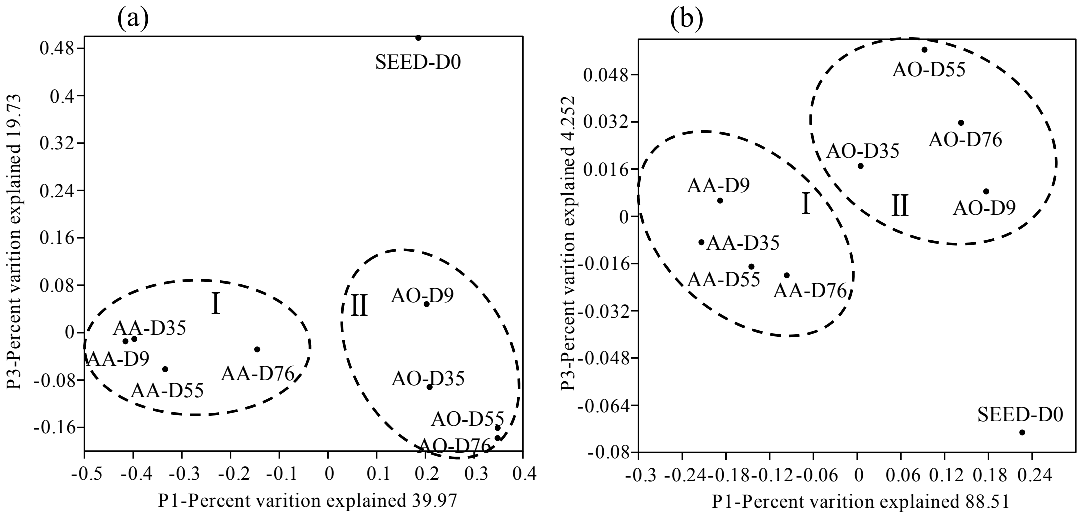


**Fig. S3.** PCoA based on Bray-Curtis distances of 9 sludge samples. (a) at 3% cutoff-OTU level; (b) at Phylum level.

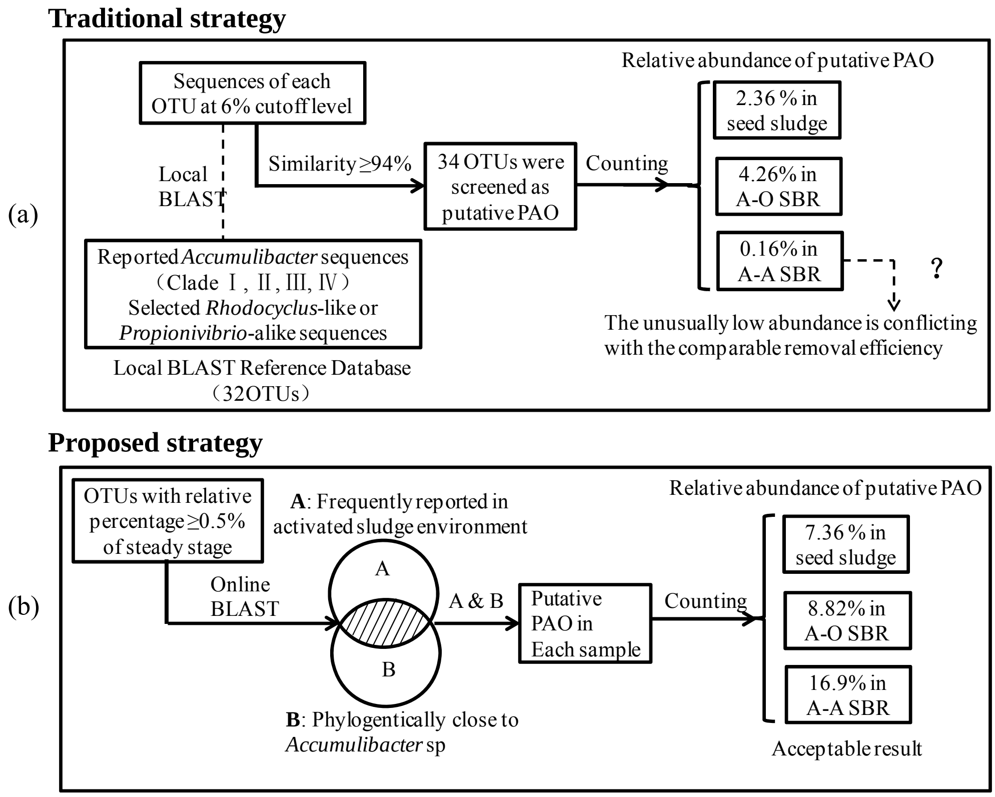


**Fig. S4.** Analysis scheme for identification of putative PAO. For identification of above-mentioned novel DPAO, another strategy emphasizing isolation source of related NCBI Genbank sequences was attempted, as illustrated by Fig.S4. Two assumptions were hypothesized for this strategy: (1) the novel DPAO should be an abundant population (with a least percentage above 0.5% or so) since too tenuous population was still incapable to explain the significant phosphorus removal efficiency. Theoretically, the situation of “vast but diverse” DPAO population should be considered, but this was conflicting with the ecological rule that population in the overlapped ecological niche could not co-exist after long time evolution. This assumption was also partially testified by the fact that more than 94% of the identified PAO in A-O SBR through traditional strategy had a relative abundance higher than 0.5%. (2) The DPAO sequence should be frequently recorded in NCBI Genbank, although they might not be correctly annotated as PAO or DPAO. Based on above two assumptions, all clustered OTUs in seed sludge, A-A and A-O sludge were separately ranked by their relative abundance. Only the abundant OTUs (relative abundance >0.5%) were further proceeded to avoid huge unnecessary work. Then the abundant OTUs were subjected to online BLAST for a comprehensive survey of the isolation source of their close relatives.

**Table S1** Ranked OTUs in A-A SBR by their relative abundances and isolation environments description of their related sequence.$^{\delta}$

| OTUs | Proportion (%) | | Similar sequence | | | Major environments |
|---|---|---|---|---|---|---|
| | A/A | A/O | Sequence NO. | Similarity (%) | Origin | |
| 1* | 16.8 | 4.56 | JQ072864 | 100 | Full-scale EBPR (12) | Widely reported in biological P-removal and wastewater treatment systems |
| | | | HQ158656 | 100 | SBR (25) | |
| 21* | 14.5 | 1.36 | HQ917038 | 100 | SBR (2) | Widely reported in water treatment systems, phylogenetically similar to *Thauera* |
| | | | HQ403231 | 100 | SBR (10) | |
| 25 | 9.01 | 7.51 | JF817650 | 100 | Microbial fuel cells (24) | Commonly reported in anaerobic conditions, soil and sediment |
| | | | HQ049856 | 93 | Oil sands tailings pond (17) | |
| 462 | 8.38 | 0.02 | FM242265 | 100 | Coastal sediment (15) | Commonly reported in anaerobic conditions, soil and sediment |
| | | | AM422228 | 98 | Sediment (7) | |
| 176 | 2.49 | 0.08 | JF817705 | 100 | Microbial fuel cells (24) | Commonly reported in microbial fuel cells |
| | | | FJ375431 | 100 | Microbial fuel cells (13) | |
| 6 | 2.35 | 0.12 | CU918778 | 100 | Anaerobic digestion of sludge (18) | Commonly reported in anaerobic conditions |
| | | | FJ66054 | 100 | Pilot-scale WWTP (19) | |
| 312* | 1.92 | 0.03 | JN090838 | 97 | EBPR SBR (9) | Widely reported in biological P-removal wastewater treatment systems |
| | | | HQ010731 | 97 | SBR (11) | |
| 806 | 1.92 | 0.02 | GU300327 | 90 | Coastal sediment (1) | Commonly reported in soil and sediment. |
| | | | AF507646 | 89 | Soil (6) | |
| 129* | 1.80 | 0.62 | FJ434606 | 100 | Bioreactor (4) | Widely reported in wastewater treatment systems and also the biological P-removal systems |
| | | | AF502223 | 100 | SBR (14) | |
| 34 | 1.62 | 0.65 | JF345459 | 100 | Citrus rhizosphere (22) | Commonly reported in soil and plant rhyzosphere |
| | | | JQ965569 | 100 | Cropped rice (5) | |
| 293 | 1.49 | 1.00 | HQ119190 | 100 | Soil (23) | Commonly reported in soil and plant rhyzosphere |
| | | | GU375391 | 100 | Soil (3) | |
| 87 | 1.44 | 2.56 | HE602881 | 100 | Caves (16) | Commonly reported in soil, sediment and occasional |

| | | | | | | |
|---|---|---|---|---|---|---|
| | | | JN107387 | 100 | Plant (8) | in wastewater treatment systems |
| 100 | 1.28 | 0.25 | JN563818 | 100 | Sea (20) | Commonly reported in soil, sediment and occasional in wastewater treatment systems |
| | | | JQ965582 | 100 | Cropped rice (5) | |
| 801 | 1.03 | 0 | HQ010842 | 93 | SBR (11) | Commonly reported in soil and wastewater treatment systems |
| | | | AY157111 | 93 | WWTP (21) | |

* The related sequences were widely reported in biological phosphorus removal wastewater treatment systems

δ OTUs with significant higher hit ratio originated from nutrient removal activated sludge environment may indicate their universal distribution and functioning in such environments, therefore they should be especially concerned in DPAO identification.

## References for Table S1


1 Brooks, D.D., R. Chan, E.R. Starks, S.J. Grayston, and M.D. Jones. 2011. Ectomycorrhizal hyphae structure components of the soil bacterial community for decreased phosphatase production. FEMS Microbiol. Ecol. 76:245-255.

2 Burgmann, H., S. Jenni, F. Vazquez, and K.M. Udert. 2011. Regime shift and microbial dynamics in a sequencing batch reactor for nitrification and anammox treatment of urine. Appl. Environ. Microb. 77:5897-5907.

3 Castro, H.F., A.T. Classen, E.E. Austin, R.J. Norby, and C.W. Schadt. 2010. Soil microbial community responses to multiple experimental climate change drivers. Appl. Environ. Microb. 76:999-1007.

4 Dafale, N., L. Agrawal, A. Kapley, S. Meshram, H. Purohit, and S. Wate. 2010. Selection of indicator bacteria based on screening of 16S rDNA metagenomic library from a two-stage anoxic-oxic bioreactor system degrading azo dyes. Bioresource Technol. 101:476-484.

5 de Souza, R., A. Beneduzi, A. Ambrosini, P.B. da Costa, J. Meyer, L.K. Vargas, R. Schoenfeld, L.M.P. Passaglia. 2012. The effect of plant growth-promoting rhizobacteria on the growth of rice (Oryza sativa L.) cropped in southern Brazilian fields. Plant Soil 360:1-19.

6 Dunbar, J., S.M. Barns, L.O. Ticknor, and C.R. Kuske. 2002. Empirical and theoretical bacterial diversity in four Arizona soils. Appl. Environ. Microb. 68:3035-3045.

7 Eller, G., P. Deines, and M. Krueger. 2007. Possible sources of methane derived carbon for chironomid larvae. Aquat. Microb. Ecol.

46:283-293.

8 Fagervold, S.K., P.E. Galand, M. Zbinden, F. Gaill, P. Lebaron, and C. Palacios. 2012. Sunken woods on the ocean floor provide diverse specialized habitats for microorganisms. FEMS Microb. Ecol. 82:616-628.

9 Gonzalez-Gil, G., and C. Holliger. 2011. Dynamics of microbial community structure of and enhanced biological phosphorus removal by aerobic granules cultivated on propionate or acetate. Appl. Environ. Microb. 77:8041-8051.

10 Guo, F., S.H. Zhang, X. Yu, and B. Wei. 2011. Variations of both bacterial community and extracellular polymers: the inducements of increase of cell hydrophobicity from biofloc to aerobic granule sludge. Bioresource Technol. 102:6421-6428.

11 Ji, Z.Y., and Y.G. Chen. 2010. Using sludge fermentation liquid to improve wastewater short-cut nitrification-denitrification and denitrifying phosphorus removal via nitrite. Environ. Sci. Technol. 44:8957-8963.

12 Lee, J.Z., S. Terry, A.J. Logan, and J.R. Spear. 2011. Microbial Dynamics of a Brewery Wastewater Treatment Works. Unpublished

13 Lefebvre, O., T.T.H. Nguyen, A. Al-Mamun, I.S. Chang, and H.Y. Ng. 2010. T-RFLP reveals high beta-Proteobacteria diversity in microbial fuel cells enriched with domestic wastewater. J. Appl. Microbiol. 109:839-820.

14 McMahon, K.D., M.A. Dojka, N.R. Pace, D. Jenkins, and J.D. Keasling. 2002. Polyphosphate kinase from activated sludge performing enhanced biological phosphorus removal. Appl. Environ. Microb. 68:4971-4978.

15 Paisse, S., M. Goni-Urriza, F. Coulon, and R. Duran. 2010. How a bacterial community originating from a contaminated coastal sediment responds to an oil input. Microb. Ecol. 60:394-405.

16 Porca, E., V. Jurado, D. Zgur-Bertok, C. Saiz-Jimenez, and L. Pasic. 2012. Comparative analysis of yellow microbial communities growing on the walls of geographically distinct caves indicates a common core of microorganisms involved in their formation. FEMS Microb. Ecol. 81:255-266.

17 Ramos-Padron E., S. Bordenave, S.P. Lin, I.M. Bhaskar, X.L. Dong, C.W. Sensen, J. Fournier, G. Voordouw, and L.M. Gieg. 2011. Carbon and sulfur cycling by microbial communities in a gypsum-treated oil sands tailings pond. Environ. Sci. Technol. 45:439-446.

18 Riviere, D., V. Desvignes, E. Pelletier, S. Chaussonnerie, S. Guermazi, J. Weissenbach, T. Li, P. Camacho, and A. Sghir. 2009. Towards the

definition of a core of microorganisms involved in anaerobic digestion of sludge. ISME J. 3:700-714.

19 Satoh, H., K. Oshima, W. Suda, P. Ranasinghe, N. Li, E.G.W. Gunawardana, M. Hattori, and T. Mino. 2013. Bacterial population dynamics in a laboratory activated sludge reactor monitored by pyrosequencing of 16S rRNA. Microbes Environ. 28:65-70.

20 Smedile, F., E. Messina, V. La Cono, *et al*. 2013. Metagenomic analysis of hadopelagic microbial assemblages thriving at the deepest part of Mediterranean Sea, Matapan-Vavilov Deep. Environ. Microbiol. 15:167-182.

21 Smith, N.R., Z. Yu, and W.W. Mohn. 2003. Stability of the bacterial community in a pulp mill effluent treatment system during normal operation and a system shutdown. Water Res. 37:4873-4884.

22 Trivedi, P., Z.L. He, J.D. van Nostrand, G. Albrigo, J.Z. Zhou, and N. Wang. 2012. Huanglongbing alters the structure and functional diversity of microbial communities associated with citrus rhizosphere. ISME J. 6:363-383.

23 Williamson, K.E., J. Kan, S.W. Polson, and S.J. Williamson. 2011. Optimizing the indirect extraction of prokaryotic DNA from soils. Soil Biol. Biochem. 43:736-748.

24 Yates, M.D., P.D. Kiely, D.F. Call, H. Rismani-Yazdi, K. Bibby, J. Peccia, J.M. Regan, B.E. Logan. 2012. Convergent development of anodic bacterial communities in microbial fuel cells. ISME J. 6:2002-2013.

25 Zhu, X., and Y. Chen. 2011. Reduction of $N_2O$ and NO generation in anaerobic-aerobic (low dissolved oxygen) biological wastewater treatment process by using sludge alkaline fermentation liquid. Environ. Sci. Technol. 45:2137-2143.

**Table S2**. Sequences and references for GAO BLAST

| Accession number | Reference |
|---|---|
| JN647912.1~JN647900.1 | Liu Z, Pruden A, Ogejo JA, Knowlton KF. Development and application of qPCR to quantify glycogen accumulating organisms (GAOs) in EBPR systems. unpublished |
| HQ341392.1~HQ341387.1 | Kim JM, Lee HJ, Lee DS, Lee K, Jeon CO. Identification of a novel subgroup of uncultured gammaproteobacterial glycogen-accumulating organisms in enhanced biological phosphorus removal sludge. Microbiology, 2011, 157 (8): 2287-2296. |
| AF361096.1~AF361089.1 | Crocetti GR, Banfield JF, Keller J, Bond PL, Blackall LL. Glycogen-accumulating organisms in laboratory-scale and full-scale wastewater treatment processes. Microbiology 2002, 148 (11): 3353-3364. |
| DQ146475.1~DQ146465.1 | Meyer RL, Saunders AM, Blackall LL. Putative glycogen-accumulating organisms belonging to the Alphaproteobacteria identified through rRNA-based stable isotope probing. Microbiology, 2006, 152 (2): 419-429. |
| AY254696.1~AY254687.1 | Beer M, Kong YH, Seviour RJ. Are some putative glycogen accumulating organisms (GAO) in anaerobic: aerobic activated sludge systems members of the alpha-Proteobacteria? Microbiology, 2004, 150(7): 2267-2275. |
